# Supplementary material for: Reoxygenation Modulates the Adverse Effects of Hypoxia on Wound Repair
Source: Int J Mol Sci. 2022 Dec 13;23(24):15832. doi: 10.3390/ijms232415832 (PMC9781139; doi:10.3390/ijms232415832)
Supplement: Supplementary file 1 [file ijms-23-15832-s001.zip › ijms-2046527-supplementary.pdf]

Supplementary Information for

Reoxygenation modulates the adverse effects of hypoxia on wound repair

Que Bai, Qian Gao, Fangfang Hu, Caiyun Zheng, Na Sun, Wenting Chen, Jinxi Liu, Yanni Zhang, Xianglong Wu*, Tingli Lu*

Key Laboratory of Space Bioscience and Biotechnology, School of Life Sciences, Northwestern Polytechnical University, Xi'an 710072, China.

E-mail addresses: lutinglixinxin@nwpu.edu.cn.

Cells culture

HUVECs were cultured in Low Glucose (LDMEM, Biological Industries) supplemented with 10% fetal bovine serum (FBS, Biological Industries), and 1% penicillin/strepto-mycin (P/S, Biological Industries). L929 were cultured in High Glucose (HDMEM, HyClone) supplemented with 10% FBS and 1% penicillin/strept-omycin. RAW 264.7 were cultured in α-minimum essential media (α-MEM, Biological Industries) supplemented with 10% FBS and 1% penicillin/streptomycin. All cells were incubated in a humidified incubator (5% CO_2) under 37°C.

ROS measurement

ROS assay kit was used fluorescent probe DCFH-DA (2′, 7′-Dichlorofluorescin diacetate) to detect the intracellular reactive oxygen species. Briefly, DCFH-DA does not fluorescence itself and can pass through the cell membrane freely. After entering the cell, DCFH-DA can be hydrolyzed by the intracellular esterase to generate DCFH. DCFH does not penetrate cell membranes, making it easy for probes to be loaded into cells. Intracellular ROS can oxidize non-fluorescent DCFH to produce fluorescent DCF. The level of intracellular ROS can be determined by measuring the fluorescence of DCF. After 24 h of normoxic and hypoxia incubation, serum-free medium (with a final concentration of 10μM DCFH-DA) was added to the cells, and the cells were incubated in dark at 37°C for 30 min. Afterwards, the cells were washed three times in serum-free medium to remove the unloaded DCFH-DA probe, and then were imaged using a inverted fluorescence microscope (MD IL HC, Germany).

MMP measurement

JC-1 is a sensitive fluorescent probe, when the MMP is high, JC-1 gathers in the matrix of mitochondria and forms polymers (J-aggregates), which can produce red fluorescence. When the MMP is low, JC-1 cannot aggregate in the matrix of mitochondria. At this time, JC-1 is a monomer and can produce green fluorescence. Therefore, the change of MMP was detected by the change of fluorescence color. After incubation for 24 h, JC-1 probe was loaded and the cells were incubated in dark at 37°C for 20 min. Afterwards, the cells were washed three times in PBS, and then were imaged using a inverted fluorescence microscope (MD IL HC, Germany).

Histology analysis

Skin tissue samples were collected on the 3th, 7th and 11th day, fixed with 4% paraformaldehyde (Seven Innovation, China), and dehydrated with gradient ethanol, and then embedded in paraffin after transparent xylene. The slices were cut into 5 μm thick for hematoxylin-eosin (H&E staining, Servicebio, China) and Masson's trichrome staining (Servicebio, China). Finally, the slides were dehydrated and sealed, and a fluorescence microscope (Nikon-80i, Japan) was placed to observe and photograph.

Immunofluorescence Staining

In order to evaluate ROS expression, the frozen slices on the 11th and 13th day were reacted with ROS staining solution (Sigma, Germany), incubate at 37°C for 30 min kept in dark place, and then coverslip with anti-fade mounting medium. To evaluate neovascularization, the fixed and frozen sections were reacted with primary antibody CD31 and alpha smooth muscle actin (α-SMA) on the 11th and 13th days. The slices were incubated with the conjugated secondary antibodies FITC conjugated goat anti-mouse IgG (Servicebio) and FITC-conjugated goat anti-rabbit IgG (Servicebio) and installed. All of the slices were observed and photographed under an inverted fluorescence microscope (Nikon Eclipse C1). Photos were analyzed with an Caseviewer.

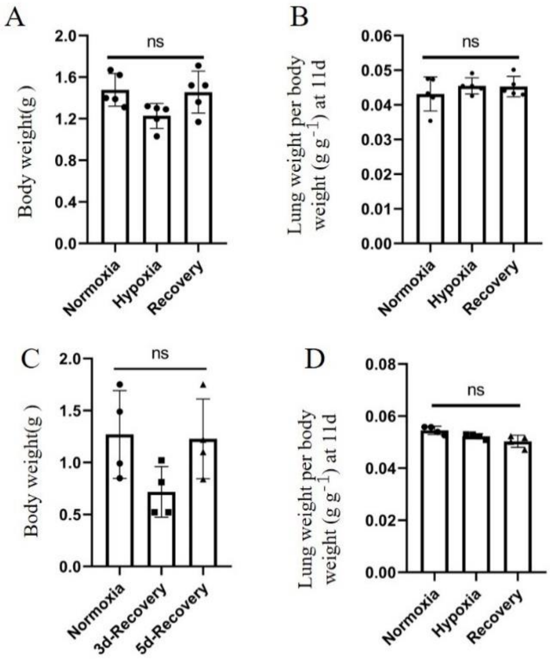

Supplementary Figure S1. Body and lung/body weight changes in mice. (A) Changes of body weight in acute wound mice. (B) Changes of lung/body weight in acute wound mice. (C) Changes of body weight in burn mice. (D) Changes of lung/body weight in burn mice. (Data are presented as mean ± S.D. n=3, n.s. no significance).

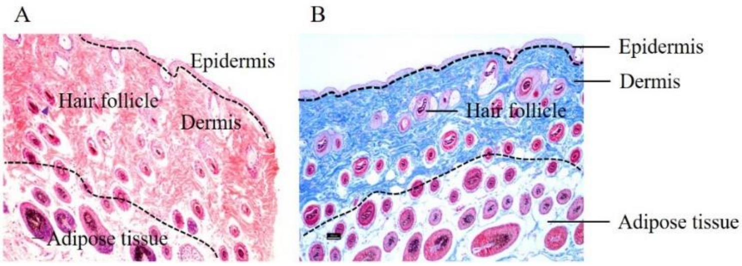

Supplementary Figure S2. Histomorphological evaluation of normal skin. (A) H&E staining of normal mice skin. (B) Masson staining of normal mice skin. Scale bar: 50 μm.

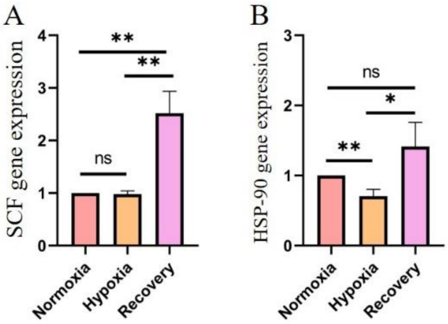

Supplementary Figure S3. The mRNA levels of HIF-1α target genes involved in wound healing were analyzed by qRT-PCR in wound tissues, cell motility (i.e. HSP-90), recruitment of CAG (i.e. SCF). (Data are presented as mean ± S.D. n=6, n.s. no significance, * indicates significant difference, * $P < 0.05$, ** $P < 0.01$).

Table S1. List of primer sequences used in RT-PCR analyses, Related to Figure 4 and Figure 9.

Table S1. The primer sequences of genes

qPCR gene	Accession	Primer sequences (5'→3')
Col I	BC007158.1	sense GGCAACAGCAGGTTCACCTACTC
		antisense GTCAGCACCACCAATGTCCAGAG
Col III	BC058724.1	sense TCTCCTGGTGCTGCTGGTCAC
		antisense TCCATGTGGTCCAACTGGTCCTC
Fibronectin	BC138421.1	sense ACAGTCCAGCAAGCAGCAAGC
		antisense TGGTGGTCACTCTGTAGCCTGTC
TNF-α	M13049.1	sense TGAGAAGTTCCCAAATGGCCTC
		antisense CTACAGGCTTGTCACTCGAATTTTG

IL-6	M20572.1	sense TAGTCCTTCCTACCCCAATTTCC
		antisense TTGGTCCTTAGCCACTCCTTC
IL-1β	M15131.1	sense GAAATGCCACCTTTTGACAGTG
		antisense TGGATGCTCTCATCAGGACAG
Arg-1	BC091158.1	sense AGGGACTGACTACCTTAAACCAC
		antisense TTCTGTCTGCTTTGCTGTGATG
IL-10	XM_012760505.1	sense GGTTGCCAAGCCTTGTCTGA
		antisense AGGGAGTTCACATGCGCCT
TGF-β	M13177.1	sense CAGTACAGCAAGGTCCTTGC
		antisense ACGTAGTAGACGATGGGCAG
VEGF	M95200.1	sense AACGATGAAGCCCTGGAGTG
		antisense TGAGAGGTCTGGTTCCCGA
CD31	BC104711.1	sense TCACCAAGAGAACGGAAGGC
		antisense CTCTTCTCGGGACATGGACG
HIF-1α	AH006789.2	sense GAATGAAGTGCACCCTAACAAG
		antisense GAGGAATGGGTTCACAAATCAG
vWF	AF152417.1	sense CAACTTGGAGCTATTGCAGGCAG
		antisense CTTTCAGAGCCAGGGAGAGAAA
α-SMA	AK137170.1	sense CCTGGCCTAGCAACACTGATT
		antisense AATTCTGTCACCCAGGGATGC
SDF-1α	BC020297.1	sense GAGAGCCACATCGCCAGAG
		antisense TTTCGGGTCAATGCACACTTG
GAPDH	NM_001411844.1	sense AAGGTCGGTGTGAACGGATTTG
		antisense TGTAGTTGAGGTCAATGAAGGGGTC
